# Fibrocyte Phenotype of ENTPD1+CD55+ Cells and Its Association with Pain in Osteoarthritic Synovium

**DOI:** 10.3390/ijms25074085

**Published:** 2024-04-06

**Authors:** Maho Tsuchiya, Yoshihisa Ohashi, Kensuke Fukushima, Yusei Okuda, Arisa Suto, Takashi Matsui, Yoshio Kodera, Masashi Sato, Ayumi Tsukada, Gen Inoue, Masashi Takaso, Kentaro Uchida

**Affiliations:** 1 Department of Orthopaedic Surgery, Kitasato University School of Medicine, Sagamihara 252-0374, Japan; 09.ma.10.ho@gmail.com (M.T.); 44134413oo@gmail.com (Y.O.); kenfu@r4.dion.ne.jp (K.F.); amidesutarere9010@yahoo.co.jp (A.T.); ginoue@kitasato-u.ac.jp (G.I.); mtakaso@kitasato-u.ac.jp (M.T.); 2 Department of Physics, School of Science, Kitasato University, Sagamihara 252-0373, Japan; okuda.yusei@st.kitasato-u.ac.jp (Y.O.); suto.arisa@st.kitasato-u.ac.jp (A.S.); matsui@kitasato-u.ac.jp (T.M.); kodera@kitasato-u.ac.jp (Y.K.); 3 Center for Disease Proteomics, School of Science, Kitasato University, Sagamihara 252-0373, Japan; 4 Department of Immunology, Kitasato University School of Medicine, Sagamihara 252-0374, Japan; msato@med.kitasato-u.ac.jp; 5 Research Institute, Shonan University of Medical Sciences, Chigasaki 253-0083, Japan

**Keywords:** knee osteoarthritis, ENTPD1, fibrocyte, pain

## Abstract

Osteoarthritis (OA) is a prevalent degenerative joint disorder characterized by cartilage erosion, structural changes, and inflammation. Synovial fibroblasts play a crucial role in OA pathophysiology, with abnormal fibroblastic cells contributing significantly to joint pathology. Fibrocytes, expressing markers of both hematopoietic and stromal cells, are implicated in inflammation and fibrosis, yet their marker and role in OA remain unclear. ENTPD1, an ectonucleotidase involved in purinergic signaling and expressed in specific fibroblasts in fibrotic conditions, led us to speculate that ENTPD1 plays a role in OA pathology by being expressed in fibrocytes. This study aimed to investigate the phenotype of ENTPD1+CD55+ and ENTPD1−CD55+ synovial fibroblasts in OA patients. Proteomic analysis revealed a distinct molecular profile in ENTPD1+CD55+ cells, including the upregulation of fibrocyte markers and extracellular matrix-related proteins. Pathway analysis suggested shared mechanisms between OA and rheumatoid arthritis. Correlation analysis revealed an association between ENTPD1+CD55+ fibrocytes and resting pain in OA. These findings highlight the potential involvement of ENTPD1 in OA pain and suggest avenues for targeted therapeutic strategies. Further research is needed to elucidate the underlying molecular mechanisms and validate potential therapeutic targets.

## 1. Introduction

Osteoarthritis (OA) stands out as a prevalent degenerative joint disorder, characterized by the progressive erosion of articular cartilage, alterations in joint structures, and chronic inflammation [1,2]. Traditionally viewed in the context of its impact on cartilage health, OA has recently been recognized as a complex condition with multifaceted pathophysiology. Pain in OA exhibits a characteristic pattern of worsening with activity and alleviation with rest, and resting pain, particularly in advanced stages, can significantly impact daily activities [3,4]. Despite its prevalence, the intricate mechanisms underlying OA-related pain, especially concerning peripheral factors, remain inadequately understood.

Recent research has expanded our understanding of OA, revealing intricate interactions among various cell types, and signaling pathways that extend beyond the joint’s articular surfaces [5,6]. Central to maintaining joint health are synovial fibroblasts, particularly CD55+ fibroblasts residing in the lining layer of the synovium [7,8,9,10]. These cells play a crucial role by forming a critical interface with the joint space and expressing high levels of lubricin, a glycoprotein, as well as hyaluronic acid, both of which are essential for joint lubrication and cartilage health [8,9]. However, in the context of OA, abnormal fibroblastic cells with a fibrotic or inflammatory phenotype become prominent, contributing significantly to the pathology of the joint disorder [11,12,13].

Among the various fibroblastic cell types found in the synovium, fibrocytes exhibit a distinct phenotype compared to resident fibroblasts, expressing markers characteristic of both hematopoietic cells and stromal cells [14]. The identification of circulating fibrocytes, considered potential precursors of synovial fibroblasts, has prompted inquiries into the characterization of fibrocytes in terms of their biological functions [15]. Additionally, fibrocytes could contribute to inflammation and pathological fibrosis across various organs [16,17,18]. Despite their significant role in inflammation and fibrosis, the specific contribution of fibrocytes to OA pathology and the identification of markers distinguishing between synovial fibroblast and fibrocyte phenotypes within the synovium remain unclear.

ENTPD1 functions as an ectonucleotidase, modulating purinergic signaling pathways by hydrolyzing extracellular ATP and ADP into AMP. ENTPD1 is present in a range of immune cells, including monocytes, neutrophils, and T cells, as well as in non-immune cells like endothelial cells and fibroblasts [19,20]. While traditionally associated with immune modulation, recent studies have highlighted its involvement in inflammatory and fibrotic conditions [21,22]. The expression of ENTPD1 in CD4+ and CD8+ T cells and B cells showed associations with disease activity indices in individuals with rheumatoid arthritis (RA) [22]. ENTPD1-expressing fibroblasts contribute to hypertrophic scar formation [21]. Given its known association with fibrosis and expression in a specific fibroblast subset, there is significant interest in exploring the expression of ENTPD1 in fibrocytes to unravel its specific role in OA pathology.

In this pursuit of a comprehensive understanding, our study delves into the phenotype of ENTPD1+CD55+ cells, aiming to unravel their intricate associations with OA pathology.

## 2. Results

### 2.1. CD55+ Population in the Osteoarthritic Synovium

**Figure 1:**
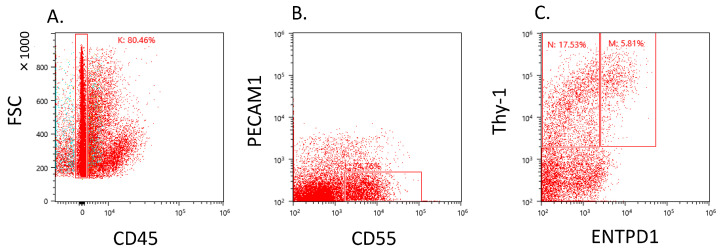
Flow cytometric analysis of ENTPD1+ and CD55+ fibroblast obtained from knee osteoarthritis patients. (**A**) CD45− gate in synovial cells. *X*-axis, CD45; *y*-axis, forward scattering (FSC). (**B**) PECAM1−CD55+ gate in CD45 negative gate. *x*-axis, CD55; *y*-axis, PECAM1. (**C**) Dot plot analysis of fibroblast population. *x*-axis, ENTPD1; *y*-axis, Thy-1.

Dot plot analysis revealed the presence of CD55+Thy-1+ cells within the CD45−–PECAM1− gate (Figure 1A,B). The CD55+Thy-1+ fraction exhibited a diverse population, including varying proportions of ENTPD1+ and ENTPD1− cells (Figure 1C).

### 2.2. Characterization of ENTPD1+CD55+ and ENTPD1−CD55+ Using LC/MS and qPCR

**Figure 2:**
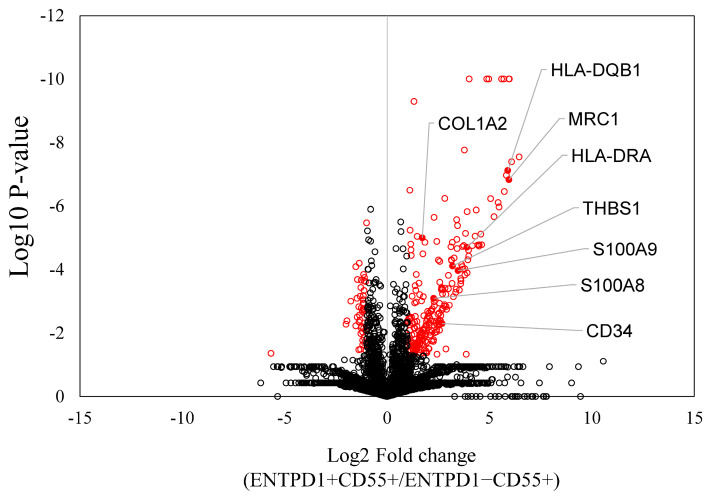
Differently expressed proteins between ENTPD1−CD55+ and ENTPD1+CD55+ cells. A volcano plot was utilized to illustrate the differentially expressed proteins between ENTPD1−CD55+ and ENTPD1+CD55+ cells. Statistically significant values (*p* < 0.05) are denoted by red circles, while non-significant values are indicated by black spots. Red dots indicate fibrocyte-related markers.

**Figure 3:**
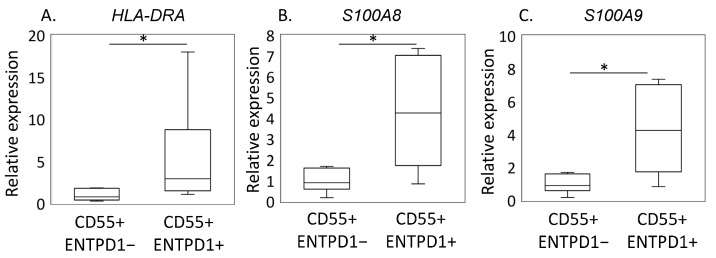
Gene expression of fibrocyte-related genes in ENTPD1−CD55+ and ENTPD1+CD55+ cells. (**A**) HLA-DRA, (**B**) S100A8, (**C**) S100A. (*n* = 5) Statistically significant values (*p* < 0.05) are indicated by *.

LC/MS analysis revealed a significant upregulation of 250 proteins in ENTPD1+CD55+ compared to ENTPD1−CD55+ fibroblasts (Supplementary Table S1). The upregulated differentially expressed proteins (DEPs) included those associated with fibrocyte-related functions, such as HLA-DRA, S100A8, S100A9, and CD34 (Figure 2). Confirming the LC/MS findings, qPCR analysis demonstrated a significantly higher gene expression of HLA-DRA, S100A8, and S100A9 in ENTPD1+CD55+ cells compared to ENTPD1−CD55+ (Figure 3). KEGG pathway analysis of the upregulated proteins in ENTPD1+CD55+ cells indicated enrichment for proteins involved in rheumatoid arthritis (e.g., HLA-DRA, ICAM1) and ECM–receptor interaction (e.g., type I collagen, Laminin subunit alpha-5, integrin-subunit alpha-6) (Table 1 and Table 2).

Conversely, 48 proteins were found to be significantly upregulated in ENTPD1−CD55+ compared to ENTPD1+CD55+ fibroblasts (Supplementary Table S2). KEGG pathway analysis of these upregulated proteins revealed enrichment for proteins associated with the HIF-1 signaling pathway (e.g., ENO1, ENO2) and Gap Junction (e.g., TUBB, TUBB2A) (Table 3).

### 2.3. Correlation between Proportion of ENTPD1+CD55+ and OA Pathology

**Figure 4:**
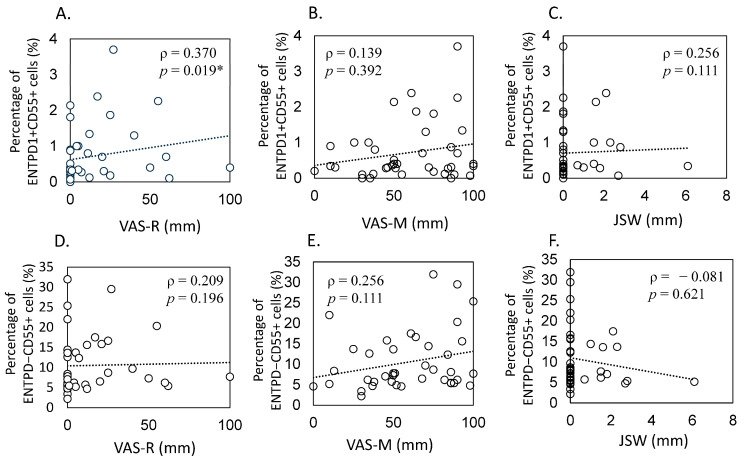
Association between the ratio of synovial ENTPD1+CD55+ and ENTPD1−CD55+ cells with radiographic joint space width and pain levels in individuals with knee osteoarthritis. The scatter plots illustrate correlations, including the proportion of ENTPD1+CD55− fibroblasts and (**A**) resting pain assessed by a visual analog scale, VAS (VAS-R), (**B**) pain during movement (VAS-M), and (**C**) radiographic joint space width (JSW). Additionally, correlations between the proportion of ENTPD1−CD55+ fibroblasts and (**D**) VAS-R, (**E**) VAS-M, and (**F**) JSW are presented. Spearman’s correlation coefficient (ρ values) was employed for statistical analysis, with statistical significance indicated by * *p* < 0.05.

Table 4 presents patient demographic and clinical features, and Figure 4A–F display scatterplots illustrating these correlations. In knee OA patients, a significant correlation was observed between the proportion of ENTPD1+CD55+ cells in the synovium and the visual analogue scale at rest (VAS-R) (ρ = 0.370, *p* = 0.019, Figure 4A). However, no correlation was found between VAS with movement (VAS-M) levels, joint space width (JSW), and the proportion of ENTPD1+CD55+ cells (VAS-M, ρ = 0.139, *p* = 0.392; JSW, ρ = 0.256, *p* = 0.111, Figure 4B,C).

Conversely, there was no correlation observed between the proportion of ENTPD1−CD55+ cells and VAS-R (ρ = 0.209, *p* = 0.196, Figure 4D), VAS-M (ρ = 0.256, *p* = 0.111, Figure 4E), and JSW (ρ = −0.081, *p* = 0.621, Figure 4F).

## 3. Discussion

The identification of 250 significantly upregulated proteins in ENTPD1+CD55+ fibroblasts compared to ENTPD1+CD55− fibroblasts highlights a distinct protein expression profile. This observation is not only indicative of the unique molecular characteristics of ENTPD1+CD55+ cells but also suggests their potential relevance in the context of OA pathology. An essential finding is the association between the proportion of ENTPD1+CD55+ cells and OA pain at rest, indicating a potential link between the molecular signature of these cells and clinical symptoms.

Recent advancements in single-cell analysis have uncovered diverse synovial fibroblast subsets in both OA and RA synovium. Single-cell RNA-seq data have categorized these subsets into distinct populations, with subsets emerging as a major player in the inflammatory regulatory signaling pathway of OA [5,13,23,24,25]. A previous study using single-cell RNA-Seq data reported that fibroblast-expressing COLLAGEN, Thy-1, thrombospondin (THBS), and CD34 were major inflammatory subsets in OA [13]. Our present proteomic analysis aligns with these findings, demonstrating that the ENTPD1+CD55+ subset expresses COLLAGEN, THBS, and CD34 compared to ENTPD1−CD55+ subsets. This consistency between transcriptomic and proteomic data strengthens the validity of our results and emphasizes the significance of the identified fibroblastic subsets in OA.

Furthermore, ENTPD1+CD55+ cells exhibit a fibrocyte phenotype, expressing markers such as HLA-DRA, S100A8, and S100A9 [26,27]. The significant presence of circulating and synovial fibrocytes in early and late RA, compared to healthy controls, has been well established in a previous study [16]. Moreover, disease activity in the biopsied joint, as assessed by ultrasound, further highlights the potential involvement of fibrocytes in the inflammatory processes associated with RA [28]. Interestingly, while fibrocytes have been extensively studied in the context of RA, our study marks a pioneering effort in investigating their contribution to OA pathology. The similarity of this phenotype to fibrocytes, which are known to be elevated in early RA, implies the potential involvement of ENTPD1+CD55+ cells in inflammatory responses in OA. Regulating synovial fibrocytes could potentially alleviate pain in OA patients, as synovial inflammation is implicated in causing osteoarthritic pain [29,30].

In the context of RA, therapeutic approaches targeting S100 proteins have shown promise [31]. Specifically, S100A8 has been considered an effective strategy for reducing inflammation and preventing cartilage and bone destruction. Treatment with anti-S100A9 antibodies has demonstrated significant improvements in clinical scores for RA patients [32]. Murine arthritis models have also demonstrated that blocking S100A8/A9 can ameliorate inflammatory processes [33], suggesting the potential for targeting S100 proteins in human arthritis patients. The expression of S100A8 and S100A9 by ENTPD1+CD55+ cells further underscores their potential contribution to OA pathology and positions them as viable therapeutic targets. This association highlights the intricate involvement of these cells in inflammatory and catabolic processes within the joint, suggesting avenues for therapeutic interventions for OA pathology.

The pathway analysis revealing enrichment in RA-related pathways, specifically involving MHC class II and ICAM1, provides intriguing insights into potential shared molecular mechanisms between RA and OA. While MHC class II molecules are traditionally associated with classical antigen-presenting cells like dendritic cells, macrophages, and B cells, fibroblast could express in MHC class II in inflammatory condition [34,35]. This unique phenomenon has been previously documented in the context of RA, where a specific subset of sublining fibroblasts expressing high levels of MHC II, specifically HLA-DRhi, was notably expanded and plays a key role in inflammation in RA synovial tissues [25,36]. The expansion of this subset was reported to be more than 15 times greater in activated RA synovial tissues [36]. Interestingly, our study identifies a similar subset of fibroblasts expressing MHC class II in the context of OA, suggesting potential convergence of the immunological pathways between these two distinct arthritic conditions. Furthermore, the presence of ICAM1+ fibroblasts, identified as key drivers of inflammation, implies a feedback loop that sustains stromal activation during fibrosis progression [37]. The expansion of this subset in OA synovial tissues, as indicated by the pathway analysis, holds implications for the pathogenesis of OA. ENTPD1+CD55+ cells may have a potential role in perpetuating the inflammatory microenvironment within the OA synovium.

Our observation of the fibrocytic population expressing key extracellular matrix (ECM) components, including collagen, laminin, THBS, and vitronectin, provides crucial insights into the molecular landscape of OA. These ECM markers play pivotal roles in defining the characteristics and functions of fibroblasts within the context of our investigation, shedding light on their involvement in maintaining tissue structure and integrity. A recent study suggested that collagen and laminin signaling predominate in OA but not RA [13]. THBS is known to modulate various cellular responses, including cell adhesion, migration, and proliferation. THBS1 expressed in fibroblasts and the THBS concentration in synovial fluid increased in OA patients compared to healthy control [38]. Moreover, the increased levels of a vitronectin fragment in the serum of OA patients compared to healthy and RA subjects highlight the relevance of fibroblasts in ECM production [39]. Understanding the intricate interplay between fibrocytes and ECM components provides valuable insights into the underlying molecular mechanisms driving OA pathology.

The literature has proposed diverse connections between pain associated with OA during periods of rest and activity in the knee and hip joints [40,41,42]. Lundblad et al. reported an association between high preoperative VAS scores for resting pain and a lower pain threshold [40]. In this context, our study introduces a novel aspect by exploring the correlation between the proportion of ENTPD1+CD55+ fibrocytic cells and VAS at rest in OA. The observed correlation between the proportion of ENTPD1+CD55+ fibrocytic cells and pain scores adds a layer of complexity to our understanding of OA pathology. It implicates these specific fibrocytes in the modulation of pain perception, potentially through their involvement in inflammatory processes or fibrotic mechanisms. Synovial fibrosis, a known factor in causing joint pain and stiffness in arthritis, could play a pivotal role in hyperalgesia in OA patients [43]. Inflammatory conditions are recognized as significant contributors to rest pain, and our study aligns with this notion, emphasizing the potential involvement of ENTPD1+CD55+ fibrocytic cells in mediating inflammation and fibrosis, contributing to the resting pain experienced by individuals with OA.

While a previous study has suggested that the deposition of CD55 on the synovium provides protection against immune complex-mediated arthritis, our current investigation did not establish a direct correlation between the proportion of ENTPD1−CD55+ cells and radiographic OA or pain scores. Tubulins, constituting essential components of microtubules, are cytoskeletal proteins belonging to the globular protein family [44]. In our study, the expression of tubulin was found to be significantly higher in ENTPD1−CD55+ cells compared to ENTPD1+CD55+ cells. Previous studies have indicated that a reduction in tubulin synthesis in the synoviocytes of rats with adjuvant arthritis could contribute to an initial step in the pathogenesis of osteoarthritis [45]. Our results suggest a potential protective role for ENTPD1−CD55+ cells in maintaining joint homeostasis, possibly mediated by tubulin expression. However, it is crucial to acknowledge the complexity of OA pathophysiology and the multifaceted roles of various fibroblast subsets within the joint. While our study hints at a potential protective role for ENTPD1−CD55+ cells through elevated tubulin expression, further investigations are warranted to unravel the underlying mechanisms and validate these findings. Future studies could explore the interplay between tubulin, ENTPD1−CD55+ cells, and other factors influencing the intricate dynamics of OA development.

The study’s findings are derived from a specific cohort of individuals diagnosed with knee OA falling within Kellgren–Lawrence (KL) grades 2–4, who underwent total knee arthroplasty (TKA). The absence of a healthy control group limits the ability to compare findings with non-OA individuals, impacting the generalizability of results. While the study focused on the expression of specific cellular markers such as HLA-DRA, S100A8, and S100A9 in ENTPD1+CD55+ cells, the functional implications of these markers and their precise role in OA pathology remain to be fully elucidated. Additionally, the small sample size of only three samples analyzed using LC-MS further limits the generalizability of our findings and may constrain the statistical robustness of the analysis. Furthermore, the absence of immunofluorescence imaging in this study hinders a comprehensive understanding of cellular interactions. Future research is warranted to delve deeper into the molecular mechanisms and functional aspects associated with these markers, as well as to explore larger sample sizes and include healthy control groups for comparative analysis.

## 4. Materials and Methods

### 4.1. Recruitment of Patients and Study Criteria

This investigation involved forty individuals diagnosed with knee OA falling within KL grades 2–4, who underwent TKA. To mitigate potential confounding factors, individuals with autoimmune diseases such as RA were excluded. Moreover, individuals with a history of significant joint trauma or prior knee joint surgery were not considered. The study received approval from the Ethics Review Board of the institution (approval number: KMEO (B22-044)), adhering to the principles of the Declaration of Helsinki. Written informed consent was obtained from all participants, encompassing their agreement for both participation and the excision of synovial tissue before TKA. TKA procedures were conducted on all participants at the institution, involving the sampling of synovial tissues from parapatellar sites.

### 4.2. Cell Sorting

Synovial cells were isolated from 3 patients for cell sorting, with three samples analyzed using LC/MS and five samples subjected to quantitative PCR. Each synovial sample was subjected to 2 h of digestion at 37 °C using a collagenase solution. Following digestion, synovial cells were treated with specific antibodies, including anti-human PECAM1 (Brilliant Violet 421), CD45 (FITC), CD55 (PE), ENTPD1 (APC/Cy7), and Thy-1 (PE-Cy7) for 45 min at 4 °C. All antibodies were purchased from BioLegend (San Diego, CA, USA). After washing twice with PBS, the synovial cells were subjected to cell sorting. The PECAM1-CD45-CD55+ENTPD1-Thy-1+ cells, referred to as ENTPD1+CD55+ cells, and PECAM1-CD45-CD55+ENTPD1-Thy-1+ cells, referred to as ENTPD1−CD55+ cells, were individually sorted using a cell sorter (MA900, SONY, Tokyo, Japan). Gating criteria were established using control samples and single-stained samples [12].

### 4.3. LC/MS Analysis

Proteins showing differential expression between ENTPD1-CD55+ and ENTPD1+CD55+ cells were identified in samples from three patients through LC-MS analysis. Following the cell sorting process, cells underwent three PBS washes and subsequent disruption using a Bioruptor sonicator (Sonicbio Co., Ltd., Samukawa, Kanagawa, Japan) for 30 min, with intervals of 30 s on and 30 s off, using a high setting in an ice water bath. For protein extraction, each batch of ≤10^5^ cells was treated with 20 µL of a PTS solution containing sodium deoxycholate, sodium lauryl sulfate, and TEAB. The sonicated mixture underwent centrifugation at 19,000× *g* for 15 min at 4 °C to separate insoluble components. The reduction of disulfide bonds occurred by incubating the supernatant with 2 µL of 200 mM Bond-Breaker TCEP solution (Thermo Fisher Scientific, Waltham, MA, USA) for 30 min at 50 °C, followed by an additional 10 min incubation on ice. The alkylation of reduced cysteine residues was achieved by introducing 2 µL of 375 mM iodoacetamide and 200 mM of TEAB for 30 min in the dark at room temperature. Subsequently, proteolytic digestion with 200 ng each of Lys-C and trypsin occurred at 37 °C for 18 h. To remove PTS from the digested sample, a solution containing 1.7% trifluoroacetic acid (TFA) was introduced, followed by centrifugation at 19,000× *g* for 15 min at 4 °C. The resulting supernatant was then subjected to desalting, and peptides were extracted using StageTips equipped with a C18 Empore disk membrane (CDS Analytical LLC, Oxford, PA, USA). Elution was performed with a solution of 0.1% TFA and 50% acetonitrile (ACN). Peptides were freeze-dried for concentration. The desiccated peptide specimen was rehydrated by employing a solution containing 0.1% formic acid (FA) and 3% ACN. This reconstitution process involved vortexing and subjecting the sample to ultrasonic agitation in a Bioruptor sonicator for 10 min, with intervals of 30 s on and 30 s off, using a high setting in an ice water bath. Prepared samples, equivalent to 1.2 × 10^3^ cells, underwent analysis using a quadrupole Orbitrap mass spectrometer (Q-Exactive, Thermo Fisher Scientific, Waltham, MA, USA) with an EASY-nLC 1000 system. Tryptic peptides were separated using a gradient of solvents A (0.1% FA) and B (90% ACN/0.1% FA) over 58 min. Eluted peptides were analyzed using a Q Exactive for data-independent acquisition (DIA)-MS. MS1 spectra were obtained at 17,500 resolutions in the range of 350–770 *m*/*z*, and MS2 spectra were collected at >200 *m*/*z* at 35,000 resolutions. DIA were performed using a previously described protocol [12]. DIA files were assessed using the DIA-NN software (v.1.8.1, https://github.com/vdemichev/DiaNN, accessed on 12 March 2024) with fixed modifications of carbamidomethyl and variable modifications of Met oxidation. Significant DEPs were determined with a *p*-value < 0.05 and log2 (fold change) > 1. The Database for Annotation, Visualization, and Integrated Discovery (DAVID) [46] was utilized for the Kyoto Encyclopedia of Genes and Genomes (KEGG) analysis [47] of the DEPs.

#### Quantitative Real-Time PCR (qRT-PCR) Analysis

To validate the results obtained through LC-MS analysis, we conducted qRT-PCR targeting a fibrocyte marker using cells from five patients distinct from the three samples subjected to LC-MS analysis. Following cell sorting, the cells underwent centrifugation, and the resultant cell pellets were dissolved using Trizol (Thermo Scientific). A spin column (Direct-zol MicroPrep kit, Zymo Research, Orange, CA, USA) was employed in subsequent RNA extraction. The quantity of RNA was determined using a spectrophotometer (Denovix, Wilmington, DE, USA). For cDNA synthesis, a total of 50 ng of RNA was utilized, employing superscript III RT^TM^ (Invitrogen, Carlsbad, CA, USA). The synthesized cDNA was then subjected to qRT-PCR analysis utilizing Syber green methods. The primer sequences are detailed in Table 5. The housekeeping gene GAPDH and the delta–delta Ct method were employed for the computation of relative mRNA expression. Additionally, the relative expression values were normalized further based on the mean values from the ENTPD1−CD55+ cell population.

### 4.4. Correlation between Proportion of ENTPD1+CD55+ Cells and OA Pathology

The progression of OA on radiographs was assessed using the KL classification [48] and by measuring radiographic JSW [49]. JSW was determined as the distance between the anterior and posterior margins at the condyle’s center, marked as the midpoint of a horizontal reference line drawn from the medial to lateral condyle margin, positioned 5 mm above the condyle’s most distal point. The femoral bone contour served as the starting point for JSW measurement, while the reference line for the tibial mid-coronal plane was established by connecting the medial and lateral edges of the tibia. Pain evaluations, including VAS-R and VAS-M, were conducted during the preoperative periods using the standard 10-point VAS, where each point represents 10 mm on the scale (ranging from 0 mm for no pain to 100 mm for the worst imaginable pain) [50]. Clinical assessments were performed at our outpatient facility a month before each surgical intervention.

To investigate the association between ENTPD1+CD55+ subsets and OA pathology, a power analysis was conducted using G*POWER3 (version G*Power 3.1.9.2), with a significance level (alpha) set at 0.05 and a power of 0.80, to ascertain the necessary sample size. The analysis indicated that 40 samples were necessary to detect a correlation between the VAS score and the proportion of ENTPD1+CD55+ cells. Consequently, a total of 40 samples were examined to assess the association between ENTPD1+ CD55+ proportions and OA pathology, considering joint space width and VAS scores. As there was only one patient in our cohort with K-L grade 2, comprising a limited number, we did not conduct a comparative analysis between K-L grades and ENTPD1+CD55+ cells.

## 5. Conclusions

Our study highlights the fibrocytic phenotype exhibited by ENTPD1+CD55+ cells, establishing a correlation between their proportion and pain at rest in OA. The distinct molecular profiles of ENTPD1+CD55+ and ENTPD1−CD55+ fibroblasts, along with their enrichment in specific pathways and their association with pain scores, lay the groundwork for targeted therapeutic strategies. This understanding may pave the way for innovative approaches to manage and treat OA, addressing both its symptomatic and disease-modifying aspects. Future research should delve deeper into the specific molecular mechanisms underpinning these correlations, offering potential therapeutic targets within the intricate landscape of OA.

## Figures and Tables

**Table 1:** DEPs in ECM-ECM receptor interaction and rheumatoid arthritis in the KEGG pathway.

Protein ID	Protein Name	Log2FC	*p*-Value	Pathway
P07711	Cathepsin L	1.596	0.001	Rheum
P16070	CD44	1.689	0.001	ECM
P08123	Collagen alpha-2(I) chain	1.720	0.000	ECM
P02458	Collagen alpha-1(II) chain	2.544	0.006	ECM
P49747	Cartilage oligomeric matrix protein	2.673	0.000	ECM
P01920	HLA-DQB1	5.901	0.000	Rheum
P01903	HLA-DRA	3.904	0.000	Rheum
P05362	Intercellular adhesion molecule 1	1.425	0.001	Rheum
P23229	Integrin alpha-6	1.122	0.008	ECM
P16144	Integrin beta-4	5.714	0.000	ECM
O15230	Laminin subunit alpha-5	3.868	0.047	ECM
P61421	V-type proton ATPase subunit d 1	1.331	0.011	Rheum
O75348	V-type proton ATPase subunit G 1	1.869	0.011	Rheum
Q9UI12	V-type proton ATPase subunit H	1.372	0.040	Rheum
Q93050	V-type proton ATPase 116 kDa subunit a1	2.142	0.002	Rheum
P04004	Vitronectin	2.557	0.002	ECM

HLA-DRA; HLA class II histocompatibility antigen, DR alpha chain, HLA-DQB1; HLA class II histocompatibility antigen, DQ beta 1 chain; Rheum, rheumatoid arthritis in KEGG pathway. ECM, ECM–receptor interaction in KEGG pathway.

**Table 2:** KEGG analysis of the upregulated proteins in ENTPD1+CD55+ cells.

Pathway	Number of Genes	*p*-Value
Systemic lupus erythematosus	21	7.0 × 10^−12^
Alcoholism	22	3.6 × 10^−10^
Lysosome	18	2.1 × 10^−9^
Shigellosis	22	5.0 × 10^−8^
Phagosome	16	6.9 × 10^−7^
Neutrophil extracellular trap formation	17	2.6 × 10^−6^
Transcriptional misregulation in cancer	17	3.0 × 10^−6^
Rheumatoid arthritis	10	1.5 × 10^−4^
Tuberculosis	13	4.2 × 10^−4^
Valine, leucine, and isoleucine degradation	7	4.9 × 10^−4^
Toxoplasmosis	10	5.5 × 10^−4^
ECM–receptor interaction	9	5.7 × 10^−4^
Complement and coagulation cascades	8	2.2 × 10^−3^
Metabolic pathways	49	2.9 × 10^−3^
*Staphylococcus aureus* infection	8	4.2 × 10^−3^

**Table 3:** KEGG analysis of the upregulated proteins in ENTPD1−CD55+ cells.

Pathway	Number of Genes	*p*-Value
Carbon metabolism	8	1.6 × 10^−7^
Biosynthesis of amino acids	6	7.2 × 10^−6^
Metabolic pathways	17	3.4 × 10^−5^
Glycolysis/glucogenesis	5	1.0 × 10^−4^
HIF-1 signaling pathway	4	7.4 × 10^−3^
Biosynthesis of nucleotide sugars	3	8.2 × 10^−3^
Amino sugar and nucleotide sugar metabolism	3	1.4 × 10^−2^
Cysteine and methionine metabolism	3	1.6 × 10^−2^
RNA degradation	3	3.4 × 10^−2^
Pathogenic *Escherichia coli* infection	4	3.6 × 10^−2^
Gap Junction	3	4.2 × 10^−2^
Salmonella infection	4	6.3 × 10^−4^
Prion disease	4	2.2 × 10^−3^

**Table 4:** Clinical characteristics of knee osteoarthritis patients.

Age (years)	74.1 ± 9.2
Sex, male/female, *n*	10/30
BMI (kg/m^2^)	27.2 ± 4.9
KL grade (2/3/4), *n*	1/10/29
JSW (mm)	2.7 ± 1.8
VAS resting pain (mm)	14.0 ± 22.7
VAS at active pain (mm)	59.2 ± 27.8

All data are reported as mean ± SD. Abbreviations: BMI, body mass index; JSW, joint space width; VAS, visual analogue scale.

**Table 5:** Sequences of the primers used in this study.

Gene	Direction	Primer Sequence (5′–3′)	Product Size (bp)
*HLA-DRA*	F	ATCCTGACCAATCAGGCGAG	124
	R	GCCTCAAAGCTGGCAAATCG	
*S100A8*	F	GACGTCTGGTTCAAAGAGTTGG	89
	R	GCCACGCCCATCTTTATCAC	
*S100A9*	F	GACTTGCAAAATGTCGCAGC	80
	R	GCCCCAGCTTCACAGAGTAT	
*GAPDH*	F	TGTTGCCATCAATGACCCCTT	202
	R	CTCCACGACGTACTCAGCG	

## Data Availability

The raw data files from all LC-MS/MS analyses have been submitted to the ProteomeXchange Consortium through the jPOST partner repository. The identifiers assigned are JPST002269 for jPOST and PXD044350 for ProteomeXchange. The data supporting the results of this study can be obtained upon request from the corresponding author, K.U.

## Supplementary Materials

The following supporting information can be downloaded at: https://www.mdpi.com/article/10.3390/ijms25074085/s1.
